# Mortality and major adverse cardiovascular events after glucagon-like peptide-1 receptor agonist initiation in patients with immune-mediated inflammatory diseases and type 2 diabetes: A population-based study

**DOI:** 10.1371/journal.pone.0308533

**Published:** 2024-08-08

**Authors:** Derin Karacabeyli, Diane Lacaille, Na Lu, Natalie McCormick, Hui Xie, Hyon K. Choi, J. Antonio Aviña-Zubieta

**Affiliations:** 1 Division of Rheumatology, Department of Medicine, University of British Columbia, Vancouver, British Columbia, Canada; 2 Arthritis Research Canada, Vancouver, British Columbia, Canada; 3 Division of Rheumatology, Allergy, and Immunology, Department of Medicine, Massachusetts General Hospital and Harvard Medical School, Boston, MA, United States of America; 4 Faculty of Health Sciences, Simon Fraser University, Burnaby, British Columbia, Canada; Army Medical University, CHINA

## Abstract

**Objective:**

To assess the risk of all-cause mortality and major adverse cardiovascular events (MACE) in patients with immune-mediated inflammatory diseases (IMIDs) and type 2 diabetes newly initiating glucagon-like peptide-1 receptor agonists (GLP-1-RAs) versus dipeptidyl peptidase-4 inhibitors (DPP-4is).

**Methods:**

We performed a population-based cohort study using administrative health data from British Columbia. Patients with an IMID (i.e., rheumatoid arthritis, psoriatic disease, ankylosing spondylitis, inflammatory bowel disease, or a systemic autoimmune rheumatic disease) and type 2 diabetes who newly initiated a GLP-1-RA or DPP-4i between January 1, 2010, and December 31, 2021 were identified using ICD-9/10 codes. The primary outcome was all-cause mortality. Secondary outcomes included MACE and its components (i.e., cardiovascular death, myocardial infarction, and ischemic stroke). Cox proportional hazard regressions were used with propensity score overlap weighting. The analysis was repeated in age- and sex-matched adults without IMIDs.

**Results:**

We identified 10,855 adults with IMIDs and type 2 diabetes who newly initiated a GLP-1-RA or DPP-4i. All-cause mortality rate was lower among initiators of GLP-1-RAs compared to initiators of DPP-4is, with a weighted hazard ratio (HR) of 0.48 (95% confidence interval [CI], 0.31–0.75) and rate difference (RD) of -9.4 (95% CI, -16.0 to -2.7) per 1000 person-years. Rate of MACE was also lower with GLP-1-RA exposure (HR 0.66 [0.50–0.88], RD -10.5 [-20.4 to -0.8]). Effect sizes were similar in adults without IMIDs.

**Conclusion:**

In patients with IMIDs and type 2 diabetes, GLP-1-RA exposure is associated with a lower risk of all-cause mortality and MACE compared to a cardioneutral active comparator.

## Introduction

Risk of cardiovascular mortality is significantly increased in many immune-mediated inflammatory diseases (IMIDs), including rheumatoid arthritis (RA) [1], psoriasis [2], ankylosing spondylitis (AS) [3], systemic lupus erythematosus (SLE) [4], ANCA-associated vasculitis (AAV) [5], and giant cell arteritis (GCA) [6]. Cardiac risk factors such as type 2 diabetes and obesity are common, well-described states of chronic low-grade inflammation that can affect the severity of underlying IMIDs [7–10]. Moreover, chronic inflammation (which may arise from autoimmune diseases or obesity) plays a key role in the pathogenesis of insulin resistance, β-cell dysfunction, and accelerated atherogenesis, highlighting a common link between autoimmunity, obesity, diabetes, and atherosclerosis [10–12]. Chronic low-grade inflammatory states arising from the interplay of one or more of these four entities may predispose affected individuals to higher rates of major adverse cardiovascular events (MACE) and premature mortality. As a result, therapies that inhibit key cytokines and pro-inflammatory pathways are being explored with aims of reducing cardiovascular risk in susceptible patients [10, 12]. In this regard, glucagon-like peptide-1 receptor agonists (GLP-1-RAs) are promising agents.

Approved for the management of type 2 diabetes and obesity, GLP-1-RAs were shown in a 2021 Cochrane meta-analysis and systematic review to reduce the risk of all-cause mortality, cardiovascular mortality, and stroke [13]. However, none of the included randomized controlled trials (RCTs) reported the effects on patients with IMIDs [13]: two studies excluded patients on systemic corticosteroids [14, 15] and one excluded patients with inflammatory bowel disease (IBD) [16]. Therefore, a gap exists in understanding whether GLP-1-RAs have similar cardioprotective effects in patients with IMIDs, who have a higher risk of cardiovascular complications due to chronic inflammation associated with their underlying condition. A scoping review on the effects of GLP-1-RAs in inflammatory arthritis and psoriasis revealed that these agents have anti-inflammatory properties through inhibition of the NF-κB pathway; however, how these effects translate clinically is limited to case reports, small uncontrolled prospective cohorts, and two small RCTs in psoriasis with conflicting results [17].

Dipeptidyl peptidase-4 inhibitors (DPP-4is) are anti-diabetic agents similar to GLP-1-RAs in that both drug classes increase GLP-1 receptor signalling [18] and were third line in British Columbia until January 2023 (at which point GLP-1-RAs became second line) [19]. While GLP-1-RAs directly stimulate the GLP-1 receptor, DPP-4is prevent breakdown of endogenous GLP-1. DPP-4is cause more modest reductions in A1c and neither induce significant weight loss nor reduce the risk of stroke or mortality [13].

We hypothesized that GLP-1-RAs may be cardioprotective for patients with IMIDs, and that patients with IMIDs (because of elevated cardiovascular risk due to chronic inflammation) may derive greater benefit than those without. To test these hypotheses, we compared the risk of all-cause mortality and new MACE (including cardiovascular mortality, myocardial infarction, and ischemic stroke) in adults with IMIDs and type 2 diabetes initiating a GLP-1-RA versus a DPP-4i, and then repeated this analysis in age- and sex-matched adults without IMIDs to compare the effect sizes.

## Materials and methods

### Source population and data

Our source population was the entire population of British Columbia (BC), Canada. Universal healthcare coverage is available for all BC residents (population ~5.2 million in 2021) [20]. We used Population Data BC, which provides access to all provincially-funded healthcare services data since 1990, including de-identified individual-level data on all outpatient medical visits [21], hospital admissions and discharges [22], and emergency room (ER) visits [23]. Population Data BC also holds the comprehensive prescription drug database, PharmaNet, which contains all pharmacy-dispensed medications for all BC residents since 1996, including dose, date, and quantity dispensed [24]. Vital statistics data on cause-specific deaths were also obtained for all patients [25].

### Study population and design

We performed a sequential propensity score (PS) overlap weighted cohort study to compare risk of all-cause mortality and MACE in new users of GLP-1-RAs or DPP-4is with IMIDs and type 2 diabetes. All GLP-1-RAs and DPP-4is approved in Canada were included (S1 Table). We included patients ≥18 years old who had an IMID (e.g., RA, psoriatic disease, AS, IBD, or a systemic autoimmune rheumatic disease [SARD] such as SLE, systemic sclerosis, Sjögren’s disease, autoimmune inflammatory myositis, GCA, AAV, Takayasu’s arteritis, and polyarteritis nodosa) and type 2 diabetes from January 1, 2010, to December 31, 2021, with at least two years of continuous enrollment in Population Data BC prior to entering the study. The diagnoses of IMID and type 2 diabetes were based on the presence of at least two physician-diagnosed International Classification of Diseases, Ninth or 10^th^ Revision (ICD-9 or ICD-10) codes (S2 Table) at least two months apart within a 2-year window for the IMID (i.e., RA, psoriatic disease, AS, IBD, or SARD) or type 2 diabetes, respectively. Case definitions for these diseases have been shown to have specificities >90% and positive predictive values between 78 and 98% in validation studies [26–30]. Our definition of psoriatic disease encompasses both psoriasis and psoriatic arthritis given the limitations of administrative data in distinguishing the two entities. Of this IMID with diabetes cohort, we identified patients whose first-ever dispensing for a GLP-1-RA or DPP-4i occurred after the diagnosis of these two diseases. The date of the first dispensing of either agent was defined as the index date. Individuals were excluded if they had been prescribed either drug class any time before the index date.

### Cohort follow-up

Follow-up began at the index date and continued until the end of the study period, end of continuous health plan enrollment, occurrence of a study outcome, or discontinuation of the initial medication (or switching to or addition of the comparator medication), whichever occurred first. A medication was considered discontinued if 60 days elapsed after the expiration date of the last prescription’s supply without a refill.

### Outcome assessment

The primary outcome was all-cause mortality (identified by death date). Secondary outcomes included MACE and its components (i.e., cardiovascular death [from any cardiac cause, defined by death date plus ICD-9 codes 390–459 or ICD-10 codes I00-I99], myocardial infarction [MI, defined by new codes of 410 or I21], and ischemic stroke [defined by new codes of 433, 434, I63, I64, I65, or I66]). Diagnostic codes for MI and ischemic stroke have high validity in administrative data with most studies yielding PPVs of ≥93% and ≥82%, respectively [31, 32].

### Covariate assessment

Covariates included sociodemographic factors (age, sex, neighborhood income quintile [measure of area-level socioeconomic status], and health authority region), comorbidities at any time from enrollment to the index date based on ICD-9 or ICD-10 codes (obesity, hypertension, MI, stroke, transient ischemic attack, congestive heart failure, ischemic heart disease, varicose veins, venous thromboembolism, chronic kidney disease, cancer, infection, and chronic obstructive pulmonary disease), diabetes-related complications (diabetic nephropathy, neuropathy, or retinopathy, and hospitalizations due to diabetes), modified Charlson Comorbidity Index for administrative data [33], medication use (other antidiabetic medications, immunosuppressives [biologics, corticosteroids, other oral and subcutaneous agents], cardiovascular medications [aspirin, statins, fibrates, diuretics, other], proton pump inhibitors, colchicine, urate-lowering therapy, opioids, non-steroidal anti-inflammatory drugs, and anticoagulants), and healthcare utilization (hospitalizations and ER visits) in the 12 months before the index date (Table 1). Diabetes and IMID duration were also included, where date of diagnosis was defined by first ICD-9/10 code (Table 1).

**Table 1:** Baseline characteristics among patients with IMIDs and type 2 diabetes initiating GLP-1-RAs or DPP-4is.

	Before overlap weighting			After overlap weighting		
Variable list	GLP-1-RA (n = 3,570)	DPP-4i (n = 7,285)	SMD	GLP-1-RA (n = 3,570)	DPP-4i (n = 7,285)	SMD
**Demographics**						
Age, mean (SD), y	58.00 (11.38)	65.74 (12.08)	0.659	60.67 (6.40)	60.67 (5.18)	<0.001
Male, n (%)	1236 (34.6)	3597 (49.4)	0.302	42.5	42.5	<0.001
Neighbourhood Income Quintile, n (%)			0.206			<0.001
1 = Lowest	684 (19.2)	1787 (24.5)		20.7	20.7	
2	704 (19.7)	1625 (22.3)		20.5	20.5	
3	694 (19.4)	1495 (20.5)		20.7	20.7	
4	781 (21.9)	1276 (17.5)		20.6	20.6	
5 = Highest	677 (19.0)	1023 (14.0)		16.7	16.7	
Unknown	30 (0.8)	79 (1.1)		0.8	0.8	
**Health Region, n (%)**			0.156			<0.001
Interior	400 (11.2)	1133 (15.6)		13.2	13.2	
Fraser	1677 (47.0)	3141 (43.1)		44.6	44.6	
Vancouver Coastal	752 (21.1)	1659 (22.8)		21.7	21.7	
Vancouver Island	563 (15.8)	955 (13.1)		15.3	15.3	
Northern	174 (4.9)	387 (5.3)		5.2	5.2	
Unknown	≤5	10 (0.1)		0.1	0.1	
**IMID, n (%)**						
RA	1367 (38.3)	2913 (40.0)	0.035	37.5	37.5	<0.001
Psoriatic disease	1487 (41.7)	2916 (40.0)	0.033	42.4	42.4	<0.001
AS	245 (6.9)	365 (5.0)	0.078	5.9	5.9	<0.001
SARD	549 (15.4)	1047 (14.4)	0.028	14.0	14.0	<0.001
IBD	528 (14.8)	1034 (14.2)	0.017	15.1	15.1	<0.001
**Mean IMID duration (SD), y**	13.1 (8.7)	13.8 (8.0)	0.082	13.3 (5.1)	13.3 (3.6)	<0.001
**Mean Charlson Comorbidity Index (SD)**	4.06 (3.26)	4.60 (3.60)	0.156	4.18 (2.02)	4.18 (1.48)	<0.001
**Comorbidities, n (%)†**						
Hypertension	2875 (80.5)	6304 (86.5)	0.162	82.9	82.9	<0.001
Myocardial infarction	423 (11.8)	1110 (15.2)	0.099	12.7	12.7	<0.001
Congestive heart failure	569 (15.9)	1535 (21.1)	0.132	16.9	16.9	<0.001
PVD	560 (15.7)	1272 (17.5)	0.048	15.5	15.5	<0.001
Ischemic heart disease	1957 (54.8)	4168 (57.2)	0.048	55.4	55.4	<0.001
Cerebrovascular accident	528 (14.8)	1416 (19.4)	0.124	15.5	15.5	<0.001
Chronic kidney disease	554 (15.5)	1461 (20.1)	0.119	16.7	16.7	<0.001
Atrial fibrillation	176 (4.9)	514 (7.1)	0.09	5.5	5.5	<0.001
Varicose veins	440 (12.3)	710 (9.7)	0.082	10.2	10.2	<0.001
Venous thromboembolism	131 (3.7)	244 (3.3)	0.017	3	3	<0.001
COPD	2457 (68.8)	4644 (63.7)	0.108	66.1	66.1	<0.001
Malignancy	2728 (76.4)	5113 (70.2)	0.141	73.1	73.1	<0.001
Infection	1101 (30.8)	2270 (31.2)	0.007	29.7	29.7	<0.001
Osteoarthritis	1369 (38.3)	3113 (42.7)	0.089	39	39	<0.001
Obesity	1205 (33.8)	980 (13.5)	0.492	22.2	22.2	<0.001
**Mean diabetes duration (SD), y**	11.2 (7.7)	12.5 (6.9)	0.188	11.4 (4.4)	11.4 (3.1)	<0.001
**Diabetic complications, n (%)**						
Diabetic nephropathy	226 (6.3)	782 (10.7)	0.158	7.5	7.5	<0.001
Diabetic retinopathy	540 (15.1)	1572 (21.6)	0.167	17	17	<0.001
Diabetic neuropathy	925 (25.9)	1536 (21.1)	0.114	23.6	23.6	<0.001
**Medications, n (%)‡**						
vAny antidiabetic medication	2578 (72.2)	6775 (93.0)	0.57	85	85	<0.001
Insulin	955 (26.8)	1030 (14.1)	0.317	22.3	22.3	<0.001
SGLT2i	596 (16.7)	559 (7.7)	0.278	14.3	14.3	<0.001
Metformin	2205 (61.8)	5994 (82.3)	0.469	75.3	75.3	<0.001
Sulfonylurea	955 (26.8)	4010 (55.0)	0.601	37.4	37.4	<0.001
Urate-lowering therapy	198 (5.5)	586 (8.0)	0.099	6.6	6.6	<0.001
Colchicine	97 (2.7)	201 (2.8)	0.003	2.5	2.5	<0.001
Aspirin	91 (2.5)	390 (5.4)	0.144	3.5	3.5	<0.001
Statin	475 (13.3)	1316 (18.1)	0.131	16	16	<0.001
Other CV medication	1508 (42.2)	3677 (50.5)	0.166	45.4	45.4	<0.001
Corticosteroid	560 (15.7)	1174 (16.1)	0.012	15	15	<0.001
Traditional NSAID	663 (18.6)	1316 (18.1)	0.013	18.2	18.2	<0.001
Cox-II inhibitor	94 (2.6)	187 (2.6)	0.004	29.8	29.8	<0.001
Fibrate	55 (1.5)	101 (1.4)	0.013	9.8	9.8	<0.001
Conventional immunosuppressant^a^	541 (15.2)	1002 (13.8)	0.04	14.2	14.2	<0.001
Biologic or JAKi^b^	292 (8.2)	333 (4.6)	0.148	6.6	6.6	<0.001
PPI	1391 (39.0)	2639 (36.2)	0.057	38.7	38.7	<0.001
Opioid	1358 (38.0)	2619 (36.0)	0.043	37.8	37.8	<0.001
Loop diuretic	391 (11.0)	892 (12.2)	0.04	11.1	11.1	<0.001
Potassium-sparing diuretic	223 (6.2)	343 (4.7)	0.068	5.4	5.4	<0.001
Thiazide diuretic	844 (23.6)	1896 (26.0)	0.055	24.9	24.9	<0.001
Nitrate	191 (5.4)	515 (7.1)	0.071	5.8	5.8	<0.001
Anticoagulant	271 (7.6)	654 (9.0)	0.05	7.8	7.8	<0.001
**Mean healthcare utilization (SD), n**‡						
Hospitalizations	0.52 (1.38)	0.63 (1.39)	0.085	0.53 (0.82)	0.53 (0.44)	<0.001
Hospitalizations due to diabetes	0.51 (0.50)	0.67 (0.47)	0.338	0.58 (0.30)	0.58 (0.21)	<0.001
Emergency room visits	0.64 (2.48)	0.68 (2.21)	0.013	0.59 (1.26)	0.59 (0.68)	<0.001

IMID, immune-mediated inflammatory disease; GLP-1-RA, glucagon-like peptide-1 receptor agonist; DPP-4i, dipeptidyl peptidase-4 inhibitor; SMD, standardized mean difference; n, number; y, years; SD, standard deviation; RA, rheumatoid arthritis; AS, ankylosing spondylitis; SARD, systemic autoimmune rheumatic disease (i.e., systemic lupus erythematosus, giant cell arteritis, ANCA-associated vasculitis, Takayasu’s arteritis, polyarteritis nodosa, systemic sclerosis, Sjögren’s disease, and autoimmune inflammatory myositis); IBD, inflammatory bowel disease; PVD, peripheral vascular disease; COPD, chronic obstructive pulmonary disease; SGLT2i, sodium glucose cotransporter-2 inhibitor; CV, cardiovascular; NSAID, non-steroidal anti-inflammatory drug; JAKi, Janus kinase inhibitor; PPI, proton pump inhibitor.

†Frequency since 1990 or enrollment.

‡Frequency during the past 1 year.

^a^Methotrexate (PO/SC), sulfasalazine, leflunomide, cyclosporine, tacrolimus, mycophenolate mofetil, azathioprine

^b^Rituximab, etanercept, infliximab, anakinra, adalimumab, abatacept, ustekinumab, golimumab, certolizumab, tocilizumab, belimumab, tofacitinib, secukinumab

### Statistical analysis

We divided calendar time into one-year blocks from 2010 (when GLP-1-RAs entered the Canadian market [34]) to 2021 (i.e., 12 blocks). Participants were allocated into one of 12 blocks based on the date they initiated a study drug. For example, participants whose initiation date fell between January 1, 2010, and December 31, 2010 would be allocated into the first time block (year 2010). Within each time block, we assembled a cohort of GLP-1-RA initiators (defined as patients who started a GLP-1-RA during that time block) and a cohort of comparator initiators who started a DPP-4i during the same time block. In each yearly time block, we calculated PS of treatment assignment (e.g., GLP-1-RA initiation) conditional on observed baseline characteristics described above and applied overlap weighting of the PS to balance baseline characteristics between the comparison groups [35]. Overlap weighting overcomes common limitations of classic PS methods by down-weighting patients at the extremes of the PS distribution rather than excluding them, improving balance and precision without sacrificing sample size [35, 36]. Individuals initiating a GLP-1-RA were weighted by the probability of not initiating a GLP-1-RA, i.e., 1-PS, and individuals initiating a DPP-4i were weighted by the probability of initiating a GLP-1-RA, i.e., PS. We assessed the balance of the distribution of baseline characteristics before and after overlap weighting by calculating the absolute standardized mean differences [37]. We calculated the weighted incidence rate (IR) for each outcome and estimated the weighted absolute rate difference (RD) between the comparison groups. We performed a Cox proportional hazard model to obtain hazard ratios (HR) and 95% confidence intervals (CI) estimating the risk for each outcome. Death was considered as the competing risk in the regression model when assessing MI and stroke as outcomes [38]. We calculated an E-value to assess the robustness of our primary outcome to unmeasured confounders [39]. Data analysis on subpopulations (e.g., RA with diabetes) were also separately conducted to assess for consistency across IMIDs.

Pre-defined sensitivity analyses excluding patients with prior MI or ischemic stroke, excluding those who discontinued either drug class within 3 months of initiation, and applying an intention-to-treat approach (following patients until the end of the study period, end of continuous health plan enrollment, or occurrence of a study outcome, whichever occurred first) were performed. Lastly, we evaluated risk of herpes zoster reactivation as a negative control outcome, expecting no difference in risk between exposure groups.

To evaluate whether patients with IMIDs derived greater benefit than those without, we assembled two additional diabetic cohorts of GLP-1-RA or DPP-4i initiators *without IMIDs* who were strata matched to our primary cohorts based on age, sex, and index date, and then we repeated our analysis in this non-IMID population.

All P values were 2-sided and P<0.05 was considered significant for all tests. All statistical analyses were performed with SAS software, version 9.4 (SAS Institute, Cary, North Carolina, USA).

No patients were involved in the design or conduct of this study. Ethics approval was obtained from the University of British Columbia’s Behavioral Research Ethics Board (H15-00887). This study followed the recommendations of the STROBE initiative for reporting observational studies in epidemiology [40].

## Results

Of the 10,855 patients included, 3,570 formed the GLP-1-RA cohort and 7,285 formed the DPP-4i comparison cohort; demographic and clinical characteristics are shown in Table 1. Before PS overlap weighting, patients initiating GLP-1-RAs had higher rates of diabetic neuropathy and obesity, as well as higher prevalence of insulin, sodium-glucose cotransporter-2 inhibitor, and biologic prescriptions. Patients initiating DPP-4is were older, more often male, and had higher prevalence of metformin and sulfonylurea prescriptions. They also had higher rates of diabetic nephropathy and retinopathy, plus higher mean Charlson Comorbidity Index and healthcare utilization. Weighted mean follow up was 1.46 and 1.88 years in GLP-1-RA and DPP-4i cohorts, respectively.

After PS weighting, the baseline characteristics between the two matched cohorts were well-balanced, with all standardized differences less than 0.10 (Table 1). The mean age was 60.67 years and the most common IMIDs were psoriatic disease (42.4%) and RA (37.5%).

### All-cause mortality

**Fig 1:**
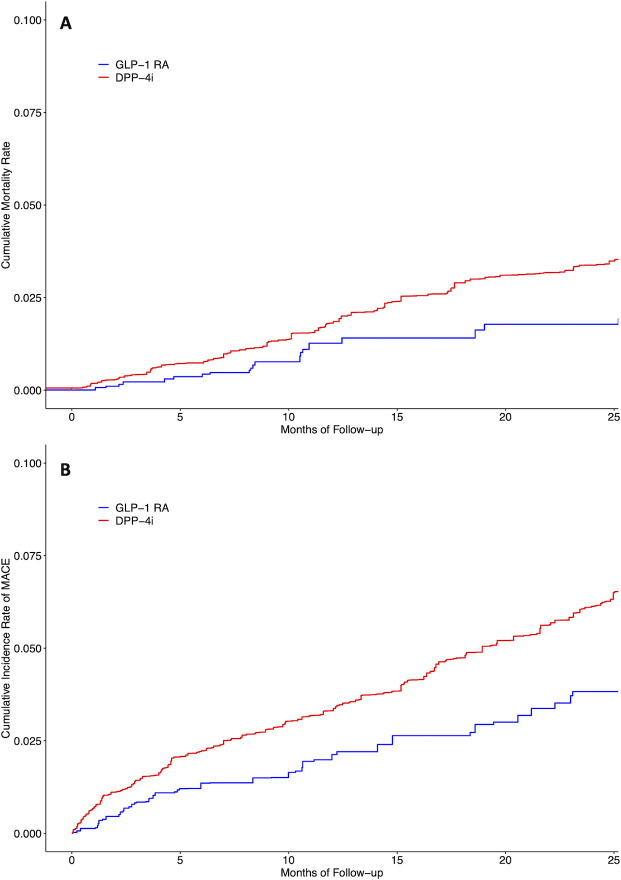
Cumulative rate of mortality (A) and MACE (B) among new users of GLP-1-RAs versus DPP-4is with IMIDs. MACE, major adverse cardiovascular events; IMID, immune-mediated inflammatory disease, GLP-1-RA, glucagon-like peptide-1 receptor agonist; DPP-4i, dipeptidyl peptidase-4 inhibitor.

Overall, there were 28 deaths among 3,570 patients with IMIDs and type 2 diabetes initiating a GLP-1-RA (8.5 per 1,000 person-years) compared to 446 deaths among 7,285 patients initiating a DPP-4i (17.9 per 1,000 person-years), yielding a weighted HR of 0.48 (CI 0.31–0.75) and RD of -9.4 (CI -16.0 to -2.7) per 1,000 person-years for all-cause mortality (Table 2). Cumulative mortality rate is presented in Fig 1A. Similar trends were observed when limiting to patients with RA or psoriatic disease (S3 and S4 Tables). In the RA-diabetes subgroup, there were 15 deaths among 1,367 new users of GLP-1-RAs compared to 219 deaths among 2,913 new users of DPP-4i (weighted IR: 9.3 versus 20.9 deaths per 1,000 person-years; HR: 0.45 [0.22–0.93], RD: -11.6 [-26.3 to 3.0]). In psoriatic disease, a trend toward reduced all-cause mortality existed, but it was not statistically significant (HR: 0.54 [0.25–1.19]). Due to small sample sizes limiting power, analysis of other subpopulations did not produce stable estimates and are therefore not reported.

**Table 2:** Mortality and MACE among patients with IMIDs initiating GLP-1-RAs or DPP-4is, after propensity score overlap weighting.

	GLP-1-RA (n = 3,570)	DPP-4i (n = 7,285)
**All-Cause Mortality**		
Event, number	28	446
Mean follow-up (years)	1.46	1.88
IR, per 1000 person-years	8.5	17.9
HR (95% CI)	0.48 (0.31, 0.75)	1.0 (ref)
RD (95% CI)	-9.4 (-16.0, -2.7)	Reference
**MACE**		
Event, number	83	632
Mean follow-up (years)	1.42	1.81
IR, per 1000 person-years	21.5	32.0
HR (95% CI)	0.66 (0.50, 0.88)	1.0 (ref)
RD (95% CI)	-10.5 (-20.4, -0.8)	Reference
**Myocardial Infarction**		
Event, number	35	313
Mean follow-up (years)	1.44	1.84
IR, per 1000 person-years	9.8	15.6
HR (95% CI)	0.62 (0.40, 0.96)	1.0 (ref)
RD (95% CI)	-5.8 (-12.5, 0.8)	Reference
**Stroke**		
Event, number	46	291
Mean follow-up (years)	1.44	1.85
IR, per 1000 person-years	10.9	14.6
HR (95% CI)	0.74 (0.50, 1.11)	1.0 (ref)
RD (95% CI)	-3.7 (-10.4, 3.1)	Reference
**Cardiovascular Death**		
Event, number	5	95
Mean follow-up (years)	1.46	1.88
IR, per 1000 person-years	1.9	3.5
HR (95% CI)	0.53 (0.20, 1.40)	1.0 (ref)
RD (95% CI)	-1.6 (-4.7, 1.6)	Reference

MACE, major adverse cardiovascular events; n, number; GLP-1-RA, glucagon-like peptide-1 receptor agonist; DPP-4i, dipeptidyl peptidase 4 inhibitor; IR, incidence rate; HR, hazard ratio; RD, risk difference; 95% CI, 95% confidence interval.

In sensitivity analyses, risk of all-cause mortality after overlap weighting was similarly lower with GLP-1-RA exposure when patients with prior MI or ischemic stroke were excluded (HR 0.42 [0.24–0.75], RD -8.5 [-14.9 to -2.0]), when patients who discontinued therapy within 3 months of initiation were excluded (HR 0.46 [0.28–0.75], RD -8.6 [-14.8 to -2.0]), and when an intention-to-treat approach (where follow up was 4.92 and 4.82 years in GLP-1-RA- and DPP-4i-exposed groups, respectively) was employed (HR 0.72 [0.60–0.86], RD -6.8 [-11.7 to -1.9]).

### Major adverse cardiovascular events

There were 83 MACE among 3,570 patients with IMIDs and type 2 diabetes initiating a GLP-1-RA (21.5 per 1,000 person-years) compared to 632 MACE among 7,285 patients initiating a DPP-4i (32.0 per 1,000 person-years). The risk of MACE was lower in new users of GLP-1-RAs compared to new users of DPP-4is (weighted HR of 0.66 [0.50–0.88], RD of -10.5 [-20.4 to -0.8] per 1,000 person-years) (Table 2). Cumulative rate of MACE is presented in Fig 1B. Weighted HRs were 0.62 (0.40–0.96) for MI, 0.53 (0.20–1.40) for cardiovascular death, and 0.74 (0.50–1.11) for ischemic stroke (Table 2). In subgroup analyses limited to patients with RA or psoriatic disease, a signal towards lower incidence of MACE was similarly observed, but this only reached statistical significance in RA (S3 and S4 Tables). In the RA-diabetes subgroup, weighted IRs for MACE in new users of GLP-1-RAs and DPP-4is were 16.2 and 40.4 per 1,000 person-years, respectively, corresponding to a HR of 0.39 (0.22–0.69) and a RD of -24.2 (-43.7 to -4.7).

Sensitivity analysis excluding patients who discontinued therapy within 3 months of initiation also illustrated a significant risk reduction (weighted HR: 0.69 [0.50–0.96]; RD: -8.5 [-17.0 to -0.1]), as did the analysis employing an intention-to-treat approach (weighted HR: 0.80 [0.69–0.94]; RD -6.4 [-12.4 to -0.4]). Sensitivity analysis excluding patients with prior MI or ischemic stroke showed fewer MACE with GLP-1-RA exposure, but this did not reach statistical significance (weighted HR: 0.72 [0.49–1.05]; RD: -7.0 [-16.1 to 2.2]).

### Outcomes in patients with and without IMIDs

Similar risk reductions in mortality and MACE were observed among patients without IMIDs initiating GLP-1-RAs versus DPP-4is in both per protocol and intention-to-treat analyses (S5 Table). Confidence intervals overlapped, indicating that effect sizes were not significantly different comparing populations with and without IMIDs. Per protocol and intention-to-treat analyses in patients without IMIDs estimated HRs of 0.34 [0.26–0.44] and 0.69 [0.63–0.76] for all-cause mortality, and 0.7 [0.6–0.8] and 0.88 [0.81–0.95] for MACE, respectively.

### Potential impact of unmeasured confounding

The E-value for our primary outcome of all-cause mortality with GLP-1-RA versus DPP-4i exposure was 3.59 (CI 2.00 to 5.91), indicating that an unmeasured confounder would need to be associated with both GLP-1-RA use and all-cause mortality by a risk ratio of 3.59 to nullify our findings.

### Control outcomes

There was no difference in herpes zoster reactivation between the two exposure groups (weighted IRs of 11.1 and 11.7 per 1,000 person-years for GLP-1-RA and DPP-4i initiators, respectively; HR 0.96 [0.65–1.43]; RD -0.6 [-1.5 to 0.3]).

## Discussion

In this large population-based cohort study comparing risk of all-cause mortality and MACE in new users of GLP-1-RAs or DPP-4is with IMIDs and type 2 diabetes, we found a 52% relative risk reduction in all-cause mortality and 34% relative risk reduction in MACE with GLP-1-RA exposure. This corresponds to nine fewer deaths and 11 fewer MACE per 1,000 person-years, respectively, supporting the hypothesis that these agents have a cardioprotective effect in this high-risk population. While insufficient power limited the number of subgroup analyses, results were directionally consistent for RA and psoriatic disease alone (each comprising about 40% of the cohort). Effect sizes were similar when compared to matched controls without IMIDs.

Elevated cardiovascular risk has been well-described in the majority of IMIDs included in this study [1–6]. While GLP-1-RAs have been shown to reduce mortality and major adverse cardiovascular events across several RCTs, patients with IMIDs have either been excluded or not reported on in these trials [13–16]. Our results fill this existing gap and suggest that GLP-1-RAs have a similar protective effect on all-cause mortality and cardiovascular morbidity in patients with IMIDs, a previously understudied but high-risk population. As DPP-4is have not been found to increase risk of death or MACE in the general population [13], we suspect our results are more likely due to GLP-1-RA-related benefit as opposed to DPP-4i-related harm. While we hypothesized that patients with IMIDs may derive greater benefit from GLP-1-RAs than the general population because of their higher baseline cardiovascular risk, this was not the case based on our data. While effect sizes in our study were larger than what has been reported in prior RCTs [13] (potentially owing to differences in study populations lending a more conservative estimate in RCTs, as well as a degree of possible unmeasured confounding in our study), they were similar between groups with and without IMIDs.

Beyond glycemic control, GLP-1-RAs may offer benefit for patients with various IMIDs through weight loss and possible adiposity-independent anti-inflammatory effects. Obesity is an inflammatory state impacting disease activity and response to therapy in several IMIDs [7]. Higher disease activity is associated with higher cardiovascular risk [41]. We therefore hypothesize that the observed reduced cardiovascular risk may be in part due to reduced adiposity leading to decreased adipokine-mediated inflammation. Clinically, whether weight loss through GLP-1 receptor agonism improves disease activity in patients with obesity and IMIDs warrants further study.

Mechanisms independent of weight loss may be at play in explaining the observed results. Basic science studies in RA, psoriasis, and IBD have demonstrated weight-independent anti-inflammatory effects of GLP-1-RAs [42–46]. One commonly described mechanism across all three diseases is GLP-1-RA-mediated inhibition of the NF-κB pathway. These agents have been found to prevent phosphorylation and breakdown of IκBα (a key inhibitory protein) in RA [43, 44, 47] and promote phosphorylation of AMPK in psoriasis (which indirectly prevents p65 nuclear translocation) [45, 48]. The role of the NF-κB pathway in atherogenesis and cardiovascular diseases is complex and remains an area of active research [49]; studies are needed to determine whether downregulation of this pathway may contribute to the lower associated risks of all-cause mortality and MACE observed in this study.

Our study has several limitations. There were imbalances in baseline covariates between the two comparison groups; however, propensity score overlap weighting was applied to address the potential confounding effect of these imbalances. After overlap weighting, new users of GLP-1-RAs and DPP-4is were perfectly balanced on all observed baseline variables. That said, residual or unmeasured confounding may still have affected study results. Capture of comorbidities relies on diagnostic codes for physician billing and therefore may be incomplete, especially with regards to obesity where sensitivity of ICD-9/10 codes are <40% [50]. Roughly 22% of our cohort had a diagnosis of obesity and we suspect this represents underreporting. That said, our E-value of 3.59 indicates that, in addition to the measured covariates that were included, an unmeasured covariate (like obesity) would need to be associated with both GLP-1-RA use and all-cause mortality by a risk ratio of 3.59 to nullify our findings. This is unlikely. As patients with obesity are at increased cardiovascular risk [51] and are more likely to receive a GLP-1-RA than a DPP-4i, the bias introduced by underreporting obesity would be conservative, favoring the null hypothesis. Smoking could not be accurately captured with our database and therefore was not included as a covariate. Uncertainty around the accuracy of the case definition for each IMID cannot be ruled out, but a strict algorithm (≥2 physician-diagnosed ICD codes ≥2 months apart within a 2-year window) was used. Such algorithms have high specificity (>90%) and moderate-to-high PPV (78–98%) across several validation studies [26–30]. Finally, while mean follow up in our per protocol analysis was between 1 and 2 years, intention-to-treat analysis (where follow-up was nearly 5 years) and sensitivity analysis excluding patients who discontinued therapy within the first 3 months yielded similar results.

This study also has strengths. We used an entire population-based database, increasing generalizability, and performed additional sensitivity analyses (e.g., excluding prior MI or ischemic stroke), enhancing the robustness of our findings. We also demonstrated no difference in our negative control outcome (herpes zoster reactivation), lending specificity to the observed effects of GLP-1-RAs.

## Conclusions

Findings from our population-based cohort study indicate that, compared with DPP-4i exposure, GLP-1-RA exposure is associated with a significantly lower risk of all-cause mortality and MACE in patients with IMIDs and type 2 diabetes, similar to what is observed in patients without IMIDs.

## Supporting information

S1 Table Included glucagon-like peptide-1 receptor agonists and dipeptidyl peptidase-4 inhibitors. (DOCX)

S2 Table Diagnostic codes used to define immune-mediated inflammatory diseases and diabetes mellitus. (DOCX)

S3 Table All-cause mortality and MACE among patients with rheumatoid arthritis and type 2 diabetes initiating GLP-1-RAs or DPP-4is, after propensity score overlap weighting. (DOCX)

S4 Table All-cause mortality and MACE among patients with psoriatic disease and type 2 diabetes initiating GLP-1-RAs or DPP-4is, after propensity score overlap weighting. (DOCX)

S5 Table Per protocol and intention-to-treat analyses of mortality and MACE among patients without IMIDs initiating GLP-1-RAs or DPP-4is, after propensity score overlap weighting. (DOCX)
