# Supplementary material for: Mortality and major adverse cardiovascular events after glucagon-like peptide-1 receptor agonist initiation in patients with immune-mediated inflammatory diseases and type 2 diabetes: A population-based study
Source: PLoS One. 2024 Aug 8;19(8):e0308533. doi: 10.1371/journal.pone.0308533 (PMC11309412; doi:10.1371/journal.pone.0308533)
Supplement: S1 Table — (DOCX) [file pone.0308533.s001.docx]

**S1 Table. List of included glucagon-like peptide-1 receptor agonists and dipeptidyl peptidase-4 inhibitors**

| Glucagon-like peptide-1 receptor agonists | Dipeptidyl peptidase-4 inhibitors |
| --- | --- |
| Liraglutide  Exenatide  Lixisenatide  Semaglutide  Dulaglutide | Alogliptin  Saxagliptin  Linagliptin  Sitagliptin |
