# Supplementary material for: Mortality and major adverse cardiovascular events after glucagon-like peptide-1 receptor agonist initiation in patients with immune-mediated inflammatory diseases and type 2 diabetes: A population-based study
Source: PLoS One. 2024 Aug 8;19(8):e0308533. doi: 10.1371/journal.pone.0308533 (PMC11309412; doi:10.1371/journal.pone.0308533)
Supplement: S2 Table — (DOCX) [file pone.0308533.s002.docx]

**S2 Table. Diagnostic codes used to define immune-mediated inflammatory diseases and diabetes mellitus**

| Diagnosis | ICD-9 code(s) | ICD-10 code(s) |
| --- | --- | --- |
| Psoriatic disease | 696.X | L40.X |
| Rheumatoid arthritis | 714.X | M05.X-M06.X |
| Systemic autoimmune rheumatic diseases (including systemic lupus erythematosus, systemic sclerosis, Sjögren’s disease, autoimmune inflammatory myositis, and systemic vasculitides) | 710.0-710.4, 446.0, 446.4, 446.5, 446.7 | M30.0, M31.3-M31.5, M32.1, M32.8, M32.9, M33.0-M33.2, M33.9, M34.X, M35.0 |
| Ankylosing spondylitis | 720.X | M45.X |
| Inflammatory bowel disease (including Crohn’s disease and ulcerative colitis) | 555.X-556.X | K50.X-K51.X |
| Diabetes mellitus | 250.X | E11.X |
