# Supplementary material for: Mortality and major adverse cardiovascular events after glucagon-like peptide-1 receptor agonist initiation in patients with immune-mediated inflammatory diseases and type 2 diabetes: A population-based study
Source: PLoS One. 2024 Aug 8;19(8):e0308533. doi: 10.1371/journal.pone.0308533 (PMC11309412; doi:10.1371/journal.pone.0308533)
Supplement: S3 Table — (DOCX) [file pone.0308533.s003.docx]

**S3 Table. All-cause mortality and MACE among patients with rheumatoid arthritis and type 2 diabetes initiating GLP-1-RAs or DPP-4is, after propensity score overlap weighting**

|  | **GLP-1-RA**  **(n=1,367)** | | **DPP-4i**  **(n=2,913)** |
| --- | --- | --- | --- |
| **All-Cause Mortality** | |  |  |
| Event, number | | 15 | 219 |
| Mean follow-up (years) | | 1.29 | 1.78 |
| IR, per 1000 person-years | | 9.3 | 20.9 |
| HR (95% CI) | | 0.45 (0.22, 0.93) | 1.0 (ref) |
| RD (95% CI) | | -11.6 (-26.3, 3.0) | Reference |
| **MACE** | |  |  |
| Event, number | | 31 | 281 |
| Mean follow-up (years) | | 1.27 | 1.72 |
| IR, per 1000 person-years | | 16.2 | 40.4 |
| HR (95% CI) | | 0.39 (0.22, 0.69) | 1.0 (ref) |
| RD (95% CI) | | -24.2 (-43.7, -4.7) | Reference |
| **Myocardial Infarction** | |  |  |
| Event, number | | 10 | 142 |
| Mean follow-up (years) | | 1.28 | 1.76 |
| IR, per 1000 person-years | | 6.7 | 18.9 |
| HR (95% CI) | | 0.37 (0.14, 1.02) | 1.0 (ref) |
| RD (95% CI) | | -12.2 (-24.8, 0.4) | Reference |
| **Stroke** | |  |  |
| Event, number | | 21 | 125 |
| Mean follow-up (years) | | 1.28 | 1.77 |
| IR, per 1000 person-years | | 10.8 | 18.3 |
| HR (95% CI) | | 0.56 (0.27, 1.17) | 1.0 (ref) |
| RD (95% CI) | | -7.5 (-21.2, 6.1) | Reference |
| **Cardiovascular Death** | |  |  |
| Event, number | | <5 | 53 |
| Mean follow-up (years) | | 1.29 | 1.78 |
| IR, per 1000 person-years | | 1.7 | 4.9 |
| HR (95% CI) | | 0.33 (0.08, 1.43) | 1.0 (ref) |
| RD (95% CI) | | -3.2 (-10.4, 4.0) | Reference |

MACE, major adverse cardiovascular events; n, number; GLP-1-RA, glucagon-like peptide-1 receptor agonist; DPP-4i, dipeptidyl peptidase 4 inhibitor; IR, incidence rate; HR, hazard ratio; RD, risk difference; 95% CI, 95% confidence interval.
