# Supplementary material for: Mortality and major adverse cardiovascular events after glucagon-like peptide-1 receptor agonist initiation in patients with immune-mediated inflammatory diseases and type 2 diabetes: A population-based study
Source: PLoS One. 2024 Aug 8;19(8):e0308533. doi: 10.1371/journal.pone.0308533 (PMC11309412; doi:10.1371/journal.pone.0308533)
Supplement: S4 Table — (DOCX) [file pone.0308533.s004.docx]

**S4 Table. All-cause mortality and MACE among patients with psoriatic disease and type 2 diabetes initiating GLP-1-RAs or DPP-4is, after propensity score overlap weighting**

|  | **GLP-1-RA**  **(n=1,487)** | | **DPP-4i**  **(n=2,916)** |
| --- | --- | --- | --- |
| **All-Cause Mortality** | |  |  |
| Event, number | | 9 | 153 |
| Mean follow-up (years) | | 1.58 | 1.88 |
| IR, per 1000 person-years | | 7.2 | 14.1 |
| HR (95% CI) | | 0.54 (0.25, 1.19) | 1.0 (ref) |
| RD (95% CI) | | -6.9 (-16.9, 3.1) | Reference |
| **MACE** | |  |  |
| Event, number | | 32 | 226 |
| Mean follow-up (years) | | 1.53 | 1.84 |
| IR, per 1000 person-years | | 22.0 | 28.6 |
| HR (95% CI) | | 0.75 (0.47, 1.20) | 1.0 (ref) |
| RD (95% CI) | | -6.6 (-22.4, 9.2) | Reference |
| **Myocardial Infarction** | |  |  |
| Event, number | | 17 | 124 |
| Mean follow-up (years) | | 1.55 | 1.86 |
| IR, per 1000 person-years | | 13.6 | 15.7 |
| HR (95% CI) | | 0.84 (0.45, 1.58) | 1.0 (ref) |
| RD (95% CI) | | -2.1 (-13.8, 9.6) | Reference |
| **Stroke** | |  |  |
| Event, number | | 15 | 94 |
| Mean follow-up (years) | | 1.55 | 1.88 |
| IR, per 1000 person-years | | 8.5 | 10.9 |
| HR (95% CI) | | 0.77 (0.37, 1.59) | 1.0 (ref) |
| RD (95% CI) | | -2.4 (-12.3, 7.4) | Reference |
| **Cardiovascular Death** | |  |  |
| Event, number | | <5 | 26 |
| Mean follow-up (years) | | 1.58 | 1.88 |
| IR, per 1000 person-years | | 1.9 | 4.6 |
| HR (95% CI) | | 0.37 (0.08, 1.66) | 1.0 (ref) |
| RD (95% CI) | | -2.7 (-7.6, 2.2) | Reference |

MACE, major adverse cardiovascular events; n, number; GLP-1-RA, glucagon-like peptide-1 receptor agonist; DPP-4i, dipeptidyl peptidase 4 inhibitor; IR, incidence rate; HR, hazard ratio; RD, risk difference; 95% CI, 95% confidence interval.
