# Supplementary material for: Mortality and major adverse cardiovascular events after glucagon-like peptide-1 receptor agonist initiation in patients with immune-mediated inflammatory diseases and type 2 diabetes: A population-based study
Source: PLoS One. 2024 Aug 8;19(8):e0308533. doi: 10.1371/journal.pone.0308533 (PMC11309412; doi:10.1371/journal.pone.0308533)
Supplement: S5 Table — (DOCX) [file pone.0308533.s005.docx]

**S5 Table. Per protocol and intention-to-treat analyses of mortality and MACE among patients without IMIDs initiating GLP-1-RAs or DPP-4is, after propensity score overlap weighting**

|  | **GLP-1-RA**  **(n=15,917)** | | **DPP-4i**  **(n=35,288)** | |
| --- | --- | --- | --- | --- |
| **All-Cause Mortality** | **Per protocol** | **Intention-to-treat** | **Per protocol** | **Intention-to-treat** |
| Event, number | 87 | 725 | 1875 | 5639 |
| Mean follow-up (years) | 1.5 | 4.98 | 1.89 | 4.92 |
| IR, per 1000 person-years | 4.7 | 12.6 | 14.2 | 18.4 |
| HR (95% CI) | 0.34 (0.26, 0.44) | 0.69 (0.63, 0.76) | 1.0 (ref) | 1.0 (ref) |
| RD (95% CI) | -9.5 (-12.0 to -6.9) | -5.8 (-7.8 to -3.6) | Reference | Reference |
| **MACE** |  | |  | |
| Event, number | 358 | 1167 | 2563 | 5686 |
| Mean follow-up (years) | 1.47 | 4.69 | 1.84 | 4.63 |
| IR, per 1000 person-years | 18.3 | 20.6 | 25.7 | 23.9 |
| HR (95% CI) | 0.70 (0.60, 0.80) | 0.88 (0.81, 0.95) | 1.0 (ref) | 1.0 (ref) |
| RD (95% CI) | -7.5 (-11.5 to -3.5) | -3.2 (-5.6 to -0.8) | Reference | Reference |
| **Myocardial Infarction** |  | |  | |
| Event, number | 194 | 660 | 1267 | 2858 |
| Mean follow-up (years) | 1.49 | 4.81 | 4.87 | 4.76 |
| IR, per 1000 person-years | 9.6 | 11.0 | 12.8 | 12.2 |
| HR (95% CI) | 0.74 (0.61, 0.90) | 0.92 (0.83, 1.03) | 1.0 (ref) | 1.0 (ref) |
| RD (95% CI) | -3.2 (-6.0 to -0.3) | -1.2 (-2.9 to 0.5) | Reference | Reference |
| **Stroke** |  | |  | |
| Event, number | 160 | 542 | 1125 | 2795 |
| Mean follow-up (years) | 1.49 | 4.84 | 1.87 | 4.78 |
| IR, per 1000 person-years | 8.3 | 11.4 | 12.1 | 9.7 |
| HR (95% CI) | 0.67 (0.55, 0.83) | 0.87 (0.78, 0.98) | 1.0 (ref) | 1.0 (ref) |
| RD (95% CI) | -3.8 (-6.5 to -1.1) | -1.7 (-3.3 to -0.1) | Reference | Reference |
| **Cardiovascular Death** |  | |  | |
| Event, number | 21 | 135 | 426 | 1250 |
| Mean follow-up (years) | 1.5 | 4.98 | 1.89 | 4.92 |
| IR, per 1000 person-years | 1.1 | 2.2 | 2.8 | 3.5 |
| HR (95% CI) | 0.39 (0.23, 0.65) | 0.64 (0.52, 0.80) | 1.0 (ref) | 1.0 (ref) |
| RD (95% CI) | -1.7 (-2.8 to -0.6) | -1.3 (-2.2 to -0.5) | Reference | Reference |

MACE, major adverse cardiovascular events; IMIDs, immune-mediated inflammatory diseases; n, number; GLP-1-RA, glucagon-like peptide-1 receptor agonist; DPP-4i, dipeptidyl peptidase 4 inhibitor; IR, incidence rate; HR, hazard ratio; RD, risk difference; 95% CI, 95% confidence interval.
